# Enhancing Evacuation Plans with a Situation Awareness System Based on End-User Knowledge Provision

**DOI:** 10.3390/s140611153

**Published:** 2014-06-24

**Authors:** Augusto Morales, Ramon Alcarria, Diego Martin, Tomas Robles

**Affiliations:** 1 Department of Telematics Engineering, Technical University of Madrid, Av. Complutense 30, Ciudad Universitaria, 28040 Madrid, Spain; E-Mails: dmartin@dit.upm.es (D.M.); trobles@dit.upm.es (T.R.); 2 Department of Topographic and Cartographic Engineering, Technical University of Madrid, Av. del Mediterráneo km 7.0 (Calle Mercator 2), 28031 Madrid, Spain; E-Mail: ralcarria@dit.upm.es

**Keywords:** evacuation plan, events conditions, actions, wireless sensor networks, publish/subscribe, situation awareness

## Abstract

Recent disasters have shown that having clearly defined preventive procedures and decisions is a critical component that minimizes evacuation hazards and ensures a rapid and successful evolution of evacuation plans. In this context, we present our Situation-Aware System for enhancing Evacuation Plans (SASEP) system, which allows creating end-user business rules that technically support the specific events, conditions and actions related to evacuation plans. An experimental validation was carried out where 32 people faced a simulated emergency situation, 16 of them using SASEP and the other 16 using a legacy system based on static signs. From the results obtained, we compare both techniques and discuss in which situations SASEP offers a better evacuation route option, confirming that it is highly valuable when there is a threat in the evacuation route. In addition, a study about user satisfaction using both systems is presented showing in which cases the systems are assessed as satisfactory, relevant and not frustrating.

## 1. Introduction

Evacuation systems are information systems involving management, operation and planning of evacuation activities. These systems have evolved into computer-based systems providing decision making in the business level, by recognizing and evaluating the threat and, at the operational level, by providing information to users for a successful evacuation.

Some weaknesses of current evacuation plans relate to the way users perceive information using fixed visual, acoustic and light signs, regardless of the emergency type, as these signs generally provide static information and evacuation routes. For example, if emergency exits are not reachable during the evacuation, individuals might not be aware of this situation until it is probably too late to react, and this fact affects the overall safety procedures and user awareness. Therefore, it is clear that a number of factors (reaction time of personnel, interaction mechanisms, labeled or static signs) could alter the evacuation plan, so they all shall be considered for a successful evacuation.

Hence, the aim of enhancing the evacuation systems is also associated to the availability of situation awareness and flexible communication capabilities with users and infrastructure. The studied emergency evacuation systems, HUENS [1], CUCEM [2], cAlert [3], Digital Building [4] and Inoue [5], provide solutions to repetitive evacuation systems in an automatic or semi-automatic way. Our Situation-Aware System for enhancing Evacuation Plans (SASEP) system provides an advantage over current emergency systems regarding people's situation awareness, and specifically in the problem identification, decision criteria in the situation of immediate evacuation. Concerning problem identification we ensure that the initial conditions, *i.e.*, information describing the status of the various actors and elements controlled by our system, are as accurate as possible, based on the experiences and current regulations. In addition, we contribute to a flexible data transmission, improving the delivery of information through publish/subscribe systems.

Related to decision criteria, SASEP is based on end-user provided business rules, which aim to minimize human errors resulting from the lack of information by providing evacuation decisions, tailored to environment's conditions and regulations.

In order to validate SASEP, the authors conducted an experimental validation involving 32 participants to address the next research questions:

(1) Which are the benefits of using SASEP to plan an evacuation route?
(2) What is the satisfaction of the people who use SASEP in an emergency situation?

The experimental validation consisted of two kinds of emergency situations proposed to the participants: the first one, where there was a threat in the evacuation route proposed by the static signs and in the second one where there was no threat in the route proposed by the static signs.

A statistical analysis of the results showed that SASEP provides a significant improvement in the quality of the routes chosen by people when there is a threat in the route proposed by static signs. Furthermore, the experimental validation shows that a special attention must be paid to the physical deployment and integration of both systems, static and dynamic; because if they work together, they can cause confusion by information overload.

The paper is structured as follows: Section 2 reviews the state of the art in decision support systems for evacuation scenarios. Section 3 explains the motivation of our work through an evacuation use case in hospitals. Sections 4 and 5 describe our proposal design and implementation respectively, and Section 6 provides an experimental validation of this approach. Finally, Sections 7 and 8 explain some results of this experimental validation and the conclusions of our work.

## 2. State of the Art

We review the state of the art in evacuation systems [6–9], considering the aspects covered in this work. These aspects include the calculus of evacuation routes, evacuation routes notification and, finally, adaptation of the system to new situations.

Related to the calculus of evacuation routes most of the solutions that may help to foresee and correctly react to evacuation scenarios are based on simulations [9]. They optimize evacuation plans for specific infrastructures, test them and detect critical points before the building is even planned. Koo *et al.* [10] and Manley *et al.* [11] define simulation models to improve evacuation procedures, considering situation awareness, as individuals with disabilities, due to physical problems (such as noise or lack of visibility) or cognitive ones (inability to understand the language in which the alerts and routes are notified or lack of familiarity with the context).

Related to user notification of evacuation routes, in order to collect and share information regarding evacuation procedures, alert notification systems are employed. Notified information ought to be appropriate to avoid panic and should state details of the emergency situation such as type, starting time, progress, affected area, and severity of disaster. The work of Malizia *et al.* [12] analyzes the most prominent alert notification systems by considering these criteria: Communication type, Source, Notification and Accessibility. This work focuses on the effectiveness of these notifications in terms of accessibility, mainly analyzing whether these systems consider people's profiles and preferences. Studied proposals [13] address effective emergency notification and evacuation by considering research in multimodal alerts, but, in our opinion, they should also consider context awareness concepts and the type of the device [14] communicating. Other works related to notifications highlight the necessity of a standard for notifications and accessibility [15]. From our point of view, these standards should take into account not only accessibility, but also, personalized messages, multimodal visualization, and various communication channels. The provision of personalized messages exploits the capability of users to express their own preferences. Multimodal visualization is related to the media by which the notifications are received by users. Some projects such as HUENS [1], CUCEM [2] and cAlert [3] consider television and radio broadcast, email, telephone, voice messages, *etc.* Supporting various communication channels facilitates the reliable delivery of a message in situations with low network coverage, and allows users to be contacted through diverse devices while maintaining a loosely coupled communication. In order to support various communication channels, a communication middleware must be provided. In previous work [16] we proposed a mobile middleware solution for continuous service invocation based on communication channels and a specific Publish/Subscribe network [17] to enable the loosely coupled communication.

The last important aspect to consider in this literature review is how evacuation systems could be adapted to new scenarios, taking into account new evacuation situations. Some works provide adaptation models that enable the fast creation of prototypes based on agent systems [18] or autonomous navigation systems [19]. These systems are often focused on route optimizations that can be based on colony algorithms [20], fuzzy logic [21] and also with algorithms inherited from communication networks. Other approaches also consider the movement of pedestrians as a homogeneous mass that behaves like a fluid flowing along corridors at a specific rate [22]. Our system differs from others, as it offers a comprehensive knowledge of the scenario in real time so it can work on top of other solutions.

Other papers, for example Aedo *et al.* [13], also take into account the importance of customized evacuation routes. Evacuation routes vary with respect to the context of each person and the characteristics of the emergency situation. Thus, an evacuation route must be customized taking into account the user's location inside the building, personal circumstances and characteristics of the situation. The problem with this approach is that it considers the customized evacuation routes as a functionality to be provided by the route notification system, without taking into account that the selection of the most appropriate route must occur in a decision support system, considering both environmental and personal aspects, as well as current regulations. Our proposed SASEP system allows broader situation awareness that leads to optimal evacuation route calculations, specifically focusing on problem identification and decision provision based on end-user provided business rules.

## 3. Importance of Situation Awareness in Evacuation Plans

The evacuation of buildings and other spaces is usually supported by static evacuation plans. These plans are obtained from the use of regulations and experiences prior to building construction. Evacuation plans can also be implemented over buildings or spaces already built due, for example, to new regulations or distribution changes. Thus, current evacuation plans associated to buildings barely take into account specific incidents people might face in an emergency [7] and the consequences of particular circumstances (e.g., fire near the emergency exit, changes in meeting points). Also, in many of these plans, evacuation signs, firefighting and emergency alert systems are not suitable for persons with special needs.

### 3.1. Motivation Scenario

This motivation scenario describes considerations regarding the two general plans [23] that can be taken during an evacuation: shelter-in-place or an imminent evacuation. We have focused on a hospital scenario in order to clarify the advantages of our system.

An Emergency Action Plan has been set up in a hospital according to Spanish recommendations [24]. This plan clearly states the chain of command, the designated person authorized to coordinate and order an evacuation, the individual roles and responsibilities of medical staff, maintenance and management staff, the threat, warning and communication actions, and the emergency response procedures in case of the actions of: imminent evacuation or shelter-in-place. Therefore, there is an evacuation baseline document that categorizes the hospital facilities according to the level of risk in the case of emergencies, their physical characteristic (e.g., building date and materials, height and accesses) and purpose (e.g., emergency rooms, operating rooms, gas deposits and kitchens). The severity of the situation is classified by the chain of command based on the input received by a set of fire, smoke, chemical or occupancy sensors, which are deployed in buildings, the evacuation baseline document and feedback provided by emergency response teams.

Suddenly a building is set on fire at midnight because of a gas leak. This building encloses facilities with operating rooms, which are only used in a fixed schedule and in rare cases out of schedule, so there is no way to accurately predict if all the rooms are occupied or empty. An evacuation platform, integrating occupancy sensors, alarms and fire sensors, can help the head of the evacuation to decide the proper measures to be taken according to the evacuation plan, and emergency response teams' capabilities. Nevertheless, it is clear that there will be a delay since the information is received by the chain of command, until it can properly decide the correct plan: to shelter-in-place or to order an imminent evacuation. All the decision must be rapidly taken based on the raw information collected on-the-fly from sensors and the expected data taken from facilities' scheduled operations. Therefore, commanding an imminent evacuation can be counterproductive if the potential threats are not well detected (e.g., the temperature has not exceeded the wall limit of fire) and the fire can be somehow controlled with the available personnel resources or fire-fighting systems. In addition, the chain of command can prematurely command the imminent evacuation of the building because of the lack of information (collected from fire-fighting systems) or wrong human perception. In this case, hospital activities might be unnecessarily disturbed along with the side-effects of starting all the evacuation procedures and shutting down the facilities, such as possible panic attacks and the impact of critical medical procedures.

Making wrong decisions product of the lack of the situation awareness also applies to the shelter-in-place plan. For example, the same fire imminently approaches to a laboratory where toxic chemicals are stored. If the chain of command underestimates (e.g., because of the lack of knowledge of regulations) or is not aware of this situation (e.g., because of poor communication capabilities), commanding the shelter-in-place plan could not be the right decision, as situations such as power or water outages can alter patients' treatments and a late reaction can affect the evacuation of physical disabled peopled and minimize their chances of survival.

In both scenarios it is clear that there are many decisions that might be taken on the fly by the chain of command; some of them based on incomplete information related to the level of the threat collected from facilities' fire-fighting and monitoring systems, information concerning the building physical characteristics, operational facts (e.g., people working, schedules), and the subjective point of view of staff, patients or emergency services. Nevertheless, in this paper we focus on the situation of imminent evacuation and present how our proposals improve evacuation plans by the means of providing better evacuation routes and user satisfaction.

### 3.2. Improving the Evacuation Plans

The situations presented in the motivation scenarios are very common and the literature [25–27], so we explain how implementing two characteristics: flexible data transmission and situation awareness, can improve the evacuation plans in hospital scenarios.

Our context situation awareness refers to the capability of proactively and reactively taking decisions based on a pre-defined set of conditions defined in the evacuation plans, and the real time information collected from the threat and the evacuation process. During the threat, which is the fire in the motivation scenario, the risks derived from the threat can also be increased. For example, if the fire approaches crowded buildings, patients can suffer from poisoning vapors or heat, fact that is not trivial in hospital environments. It means that transforming these conditions to tangible actions in evacuation systems and human's awareness improve the reactive decisions that must be taken during the evacuation. If all the preventive policies and countermeasures to deal with evacuation hazards are clearly ruled and triggered, no additional time needs to be spent by the chain of command in the process of evaluating the risks. As an example, if a fire event is located close to a gas system and exceeds the wall capacity of resisting heat, this condition could require the action of an immediate evacuation of the building, so specialists in gas leaks must be immediately informed, electrical and gas systems must be shut down; however, if the fire does not exceed a given temperature, the evacuation action could be to shelter-in-place, so different procedures must be followed. Therefore, having these kind of pre-defined action loaded into the evacuation system can help to quickly realize what are the current and future procedures that follow the fire (e.g., to take the less contaminated evacuation route and to choose a meeting point) as well as to proactively take some decisions regarding the evacuations systems (e.g., to turn on the emergency lights and audible alerts) or the building itself (e.g., to shut down the gas feed pipes and air system).

Having better situation awareness also provides advantages to humans, for example medical and safety coordinators can focus on providing support to people rather than paying attention to the evacuation procedures that are not clearly commanded. During the emergency many reactive decisions shall be taken, for example to move patients, disconnect medical devices and close windows, so the more personalized information is provided, the more chances of survival people have; and providing a broader situation awareness to arriving emergency services is a critical fact [27] for achieving a faster and correct evacuation plan. Therefore, the evacuation systems should allow interacting with humans and the existing evacuation infrastructure (e.g., communication systems, route signs, smoke sensors and mechanical actuators) in order to have the complete view of the hazards, so they can plan the proper measures.

We refer to flexible data transmission as the capability of the evacuation system to plug in heterogeneous information sources (e.g., heat sensors) while decoupling the evacuation specific actions (e.g., to turn on emergency lights). A well-known weakness [28–30] of current evacuation infrastructure is the fact that the information given, based on labeled signs, acoustic and/or illuminated alerts, is displayed in a static way, regardless of the emergency situation. For example, if one of the emergency exits cannot be used during the evacuation, people are unaware of this until the very last moment, and this situation will probably lead to a late reaction and panic. This non-flexible way to perform the evacuation clearly affects people's safety as it assumes a lack of variance when it is evident that there are plenty of different conditions that can alter its progress, such as structural damages, time/date, evacuation orders or even the disability level of the person. Therefore, a flexible data transmission can contribute to a better situation awareness as data from different contexts can reach the chain of command.

Currently, external and emergency response teams can be informed through phone calls or the activation of emergency switchboards, and more recently research work has demonstrated the advantages of interacting using mobile devices [12,13]. To the best of our knowledge mobile devices have only been used to communicate between people and safety staff, an no prior efforts have been made to take advantage of their potential regarding their capability to extend the evacuation system in real time. For example, if a fire reaches a radioactive waste facility, emergency staff could use specific radioisotope sensors to detect whether it is feasible or not enter to the facility, depending on expected exposure levels and safe limits. In a worst case scenario, the radiation limits could trigger the imminent evacuation of nearby hospital buildings, so the availability of this information can improve the reaction time of the chain of command.

### 3.3. Recommendations for Evacuation Plans in Hospitals

Evacuation plans may vary depending on different countries' regulations and recommendations. So, in the case a specific event occurs (e.g., a fire or smoke in a floor) and depending on specific conditions (e.g., in a crowded or empty facility), the actions might be very different (e.g., to initialize a vertical evacuation or immediately start the fire extinguisher system). For example in Spain, depending on the hospital size, it is recommended [24] to classify hazards according to the purpose, building types, number of patients. So decisions taking by the chain of command must take into consideration these factors. In United States, there are similar recommendations [31] to mitigate events according to the hazard, define different healthcare roles and actions, and chain of command information policy. In Australian [32], specific site-to-site procedures are used to respond to fire conditions, in addition to specific commands that should be known by emergency teams. Thus, even if countries' regulations define similar hazards, the specific events, conditions and actions resulting of these regulations can vary, so the more flexible is the system is the better compliance it will provide to the singular needs of the evacuation plans.

Finally, our proposed SASEP system tackles the problem of technically supporting specific events, conditions and actions that can be taken into account in the design and execution of evacuation plans, and the overall situation awareness.

## 4. System Design

In this section, we explain the different components comprising SASEP. We describe the architecture that support the execution of the situation awareness model according to the existing hardware and software resources and the communication between peopled involved in the evacuation plan. Afterwards, the main two subsystems are described, the Rule Provisioning Module and the communication subsystem, enabling the communication flow between peopled involved in evacuation plans.

### 4.1. Overall Architecture of the Situation Aware System for Enhancing Evacuation Plans

**Figure 1.**
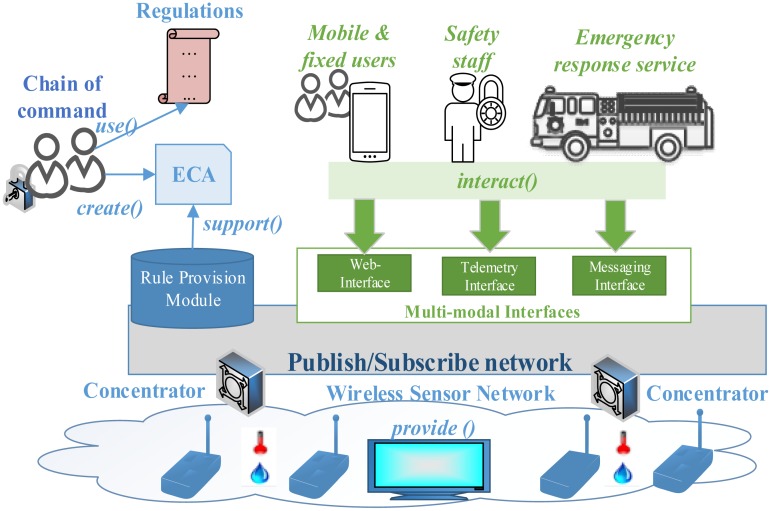
Overall architecture of SASEP.

The SASEP proposal includes various functional components, represented in Figure 1, so we list its modules and their functionalities:

The **Publish/Subscribe network** manages the delivery of information to subscribers and provides interfaces for publishers. Publishers are elements that generate information in the system (e.g., sensor readings, alerts, messages generated by the chain of command or safety staff). Subscribers are devices interested in information produced by publishers (e.g., actuators, emergency services, users). Brokers put in contact publishers and subscribers by delivering the events that contain information, this while maintaining the time, space and sync communication decoupling between publishers and subscribers.

The underlying protocol used to build the network is Message Queue Telemetry protocol (MQTT) [33], where topics identify these events. Topics are hierarchically organized in domains, shared by all the network elements, and administratively filtered by brokers. This hierarchy depends on the level of organization of the information that will be produced and the physical (e.g., floor types, halls), networking (e.g., sensors, networks), or human (e.g., staff roles) resources of the environment (e.g., a school and hospital). We use a topic domain shared by all the network participants and divided into namespaces. Topics are published in namespaces in order to receive events in the appropriate language and ensure that only compatible events are pushed by brokers. Each topic in a topic namespace (tns) can have zero or more child topics and a child topic can itself contain further child topics. A topic without a parent is termed a root topic. We use the forward slash (/) character to indicate a “child of” relationship. For example, the tns1:etsit_building/floor_1/hall_1/sensor/temperature refers to a specific sensor reading exception, subset of the parent topics hall_1,floor_1,etsit_building, in the namespace tns1.

This approach supports transformation and aggregation of topics. It is possible to construct configurations (using intermediary brokers) where the topic, an interested “subscribes to” differs from the topic under an entity “publishes”. Thus, the broker, acting in line with administratively-defined rules, receives the messages, which can be automatically triggered events (derived from the event, conditions and actions set up), matches and notifies the corresponding subscriber (e.g., fireman and police). It is possible for participants of the Pub/Sub network to define additional topics based on existing topics without requiring coordination with the participant responsible of creating the topics that are being built on. This is useful for cases where it is necessary to publish new sensors' readings, or interact with unknown actuators. This solution is compatible with WS-Topics [34], which presents a set of “items of interest for subscription” in Web service environments, and it has been extended to be aligned to a non-WS environment. The data payload of events follows a JSON format.

Regarding the inter-broker communication we employ a gossip-based routing [35] in order to provide network resilience, in case of failure of brokers; so, all the events always have an alternative route to get to brokers. A broker receives an incoming event, waits for a given time, selects the subset of brokers (fanout) to which the event is route, and then gossips the event.

The **wireless sensor network** (WSN) provides access to sensors and actuators deployed over buildings. Sensors and actuators are supported in wireless nodes integrated with the overlaying Pub/Sub network using concentrators (which perform as front-end MQTT clients), so concentrators receive/publish event, correlate event's topics with WSN identifiers and forwards events being published or received from/to the Pub/Sub network back and forth.

Our system provides three **multi-modal interfaces**: web-interface, telemetry interfaces and messaging interfaces in order to enable the interaction among users, actuators and sensors being deployed in the WSN, and receive alarms and notifications. First, the web interface provides real-time access to sensors and actuators using a web-browser. A Pub/Sub Proxy (PSP) implements this web-interface using Web-sockets [36], along with additional MQTT interfaces, HTTP-REST based interfaces that allows forwarding sensors and actuators' events from/to the Pub/Sub network. Second, the telemetry interface is implemented by brokers that accept subscriptions and events from clients. This interface complies with the MQTT protocol. Third, the messaging interfaces is based on the Extensible Messaging and Presence Protocol (XMPP) [37] and allows clients implementing this protocol to be part of a messaging infrastructure and exchange real-time messages, where a messaging central node (MCN) manages membership statuses and implements a HTTP REST-based client that allows receiving info (e.g., sensors readings and alarms) from the RPM and pushing it to XMPP clients.

The **Rule Provisioning Module** (RPM) uses the strategy of rule-based composition by mashup interconnection, which allows users (e.g., the head of the evacuation, safety staff, or emergency response services) to create and run emergency procedures based on data collected from sensors, or messages created by users, which are later evaluated inside the framework and then trigger actions in environment capabilities such as actuators and messaging services. Access to these capabilities is encapsulated in atomic interfaces. The description of these capabilities and the graphical representation of the component are designed and implemented by experts in the atomic interfaces provision step.

### 4.2. Situation Awareness in SASEP

Safety staff requires tools for improving the situation awareness by means of the realization of the decisions needed to be taken according to the real time information collected from the threat, the evacuation process and regulations. Our rule provisioning module allows end-users to generate tailored services that reflect decisions and actions for a specific situation. We based on a previous work [38], in which we developed a prosumer framework enabling end-user service composition. Our module contributes to situation awareness as it provides a web interface enabling mashup composition based on ECA rules, so that end-users can customize the behavior of the system. This composition paradigm describes the composition of emergency evacuation procedures by providing three related components: Event (E), as an occurrence triggering the rule execution, for example, e.g., temperature sensor reading; condition (C); logic statement that can be evaluated to “true” or “false”, e.g., temperature value higher than a threshold; and action (A), describing the realization of a certain action if the condition is evaluated to true, e.g., to send an immediate evacuation sign.

These requirements are checked for inconsistencies, such as circular dependencies or constraint violations through a validator that we defined in a previous work [39]. This validator is invoked when the head of the emergency saves the created or adapted procedure.

Taking back the motivation scenario of Section 3, the rule provisioning module allows the chain of command to plan the evacuation by taking into consideration, for example, a fire is detected in a specific laboratory where toxic chemicals are stored, if the temperature surpassed the wall resistance level then, the action will be to immediately evacuate and power off gas and electrical services in the whole facility and near buildings; however if the temperature remains below the wall resistance level, the gas and electrical services will be maintained. The same provision module supports a shelter-in-place situation, for example, if the temperature after does not surpass the wall resistance level, the rule provision module can trigger the action of sending a pre-defined message (defined by chain of command), with actions safety personnel must carry out (e.g., to keep a door closed).

### 4.3. Communication Workflow

**Figure 2.**
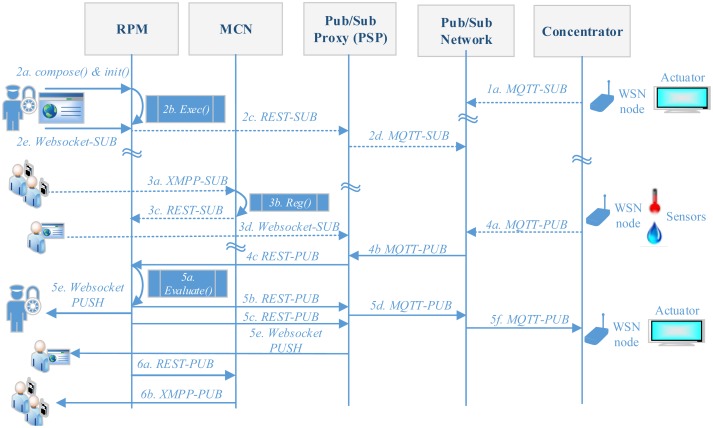
Communication workflow.

This section details the communication flow of the data transmission system. To explain this communication flow we propose an example of evacuation rule, which consists of receiving temperature and humidity sensors readings (*event*), evaluating a configured threshold (*condition*) and depending on this result, sending a tailored alarm to mobile and fixed registered users and modifies actuators' statuses (*action*), according to each one of the software implementation of the multi-modal interfaces and the RPM. Figure 2 shows the communication workflow. First, the concentrator must be subscribed (1a) to the Pub/Sub network on behalf of all the WSN nodes it supports by using all the topics that identify actions in actuators. An authorized user part of the chain of command composes and initializes (2a) the execution of the rule-based procedures using its web browser. As the rule execution (2b) requires sensors' inputs the RPM subscribes (2c) to PSP, which in turn subscribes to the Pub/Sub network. The authorize user also registers (2e) to the RPM to monitor actions. At this point registered users can make use of the multimodal interfaces to receive alarms. Mobile registered users subscribe (3a) to the same alarms, using the messaging interfaces, so the MCN registers (3b), adapts and forwards (3c) this alarm request to the RPM. Fixed registered users also subscribes (3d) to the PSP using their web-browser. After a while, the concentrator collects and pushes (4a) the sensors' data from the WSN nodes to the Pub/Sub network. Then, data are forwarded (4b) to the PSP, which later on encapsulates (4c) the payload of the MQTT-PUB message into a new REST-based publish event that reaches the RPM. At this point the RPM evaluates the existing rules (5a), so in the case the rule is hit, the RPM sends (5b) a REST-based event to the PSP which include the new actuators' statuses; then, the latter encapsulate (5d) this information into a MQTT-PUB messages that reaches (5f) the concentrator which then applies the new statues of actuators. As part of the hit rule, the RPM must send a tailored alarm, so it triggers additional multi-modal interfaces. First, the RPM sends (5c) the alarm to the PSP, which then pushes it (5e) to fixed registered users using their web-browser. Second, it pushes (6a) the same alarm to the MCN, using a REST message. Then, the MCN encapsulates the alarm into a XMPP message that is later received (6b) by mobile registered users.

## 5. Implementation and Deployment

This section explains the implementation of the SASEP and its deployment in a hospital environment.

### 5.1. Software/Hardware Implementation

**Figure 3.**
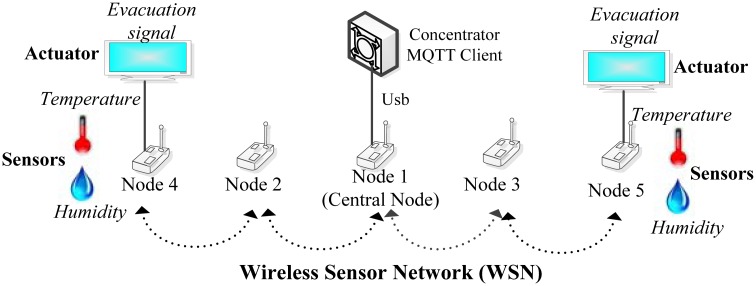
Integration of sensors and actuators.

In this section we describe the implementation of SASEP by describing each of its components. Related to the wireless sensor network, all the hardware developments that integrate sensors and actuators have been designed from scratch; it includes the printed circuit board and electronic design. The core processor of a WSN node is a low-power OMAPL127 (DSP+ARM) [40] of Texas Instruments. Nodes use the chip PSOC3 [41] of Cypress Semiconductor. The communication between WSN nodes is made following the 802.15.4 standard and a topology based on trees, where root nodes perform as data proxies between the WSN and concentrators. Nodes have been provided with rechargeable USB batteries. Concentrators and root nodes are connected using an USB interface managed by a middleware we developed in C++. The concentrator runs Montavista Linux and implements as a MQTT client developed in Java, using open-source libraries [42], together with a Java Native Interface (JNI) which enables the communication between the middleware and the MQTT client. Figure 3 shows the wireless sensor network.

The Publish/Subscribe network fully relies on the MQTT protocol including inter-broker communication. Two brokers are implemented using a modified version of the java-based Moquette broker [43], in order to support the gossip-based routing and implement the transformation and aggregation of topics explained in Section 4. We have also set up an additional mobile broker as a proof-of-concept for extending the coverage of the Publish/Subscribe network and monitoring events.

Regarding the multi-modal interfaces, the PSP has been developed in Nodejs [44], which allows clients interacting in real-time with the WSN using Websockets, so it is the front-end for fixed safety staff, and emergency staff (upon previous login). It provides a web interface that shows a map of pre-loaded locations and allows collecting information from sensors and modifying actuators' states. It also implements a MQTT client. Concerning the MCN, we have used the open-source Openfire 3.8 server [45] to offer messaging interfaces based on the XMPP protocol. We have set up three groups of standard staff, safety staff and emergency staff with the corresponding persistent chat rooms. All the software components run in two Ubuntu 12.04 32 bits machines. The concentrator and the first broker instance run in a different machine than the PSP, the RPM, the MCN and the second broker instance.

In our system, actions are generic in the way that the system supports notifying tailored messages to registered users which can make use of different fixed or mobile device (according to the communication workflow shown in Figure 2). Hence, the specific action is defined by the rule creator which can, for example, choose to automatically send a message that includes commands for both situations: shelter-in-place (e.g., to maintain all doors closed) and imminent evacuation (e.g., to take a specific route). The notification process of participants is carried out using a XMPP interface based on Asmack libraries [46] for Android, and a web browser interface provided by the PSP.

**Figure 4.**
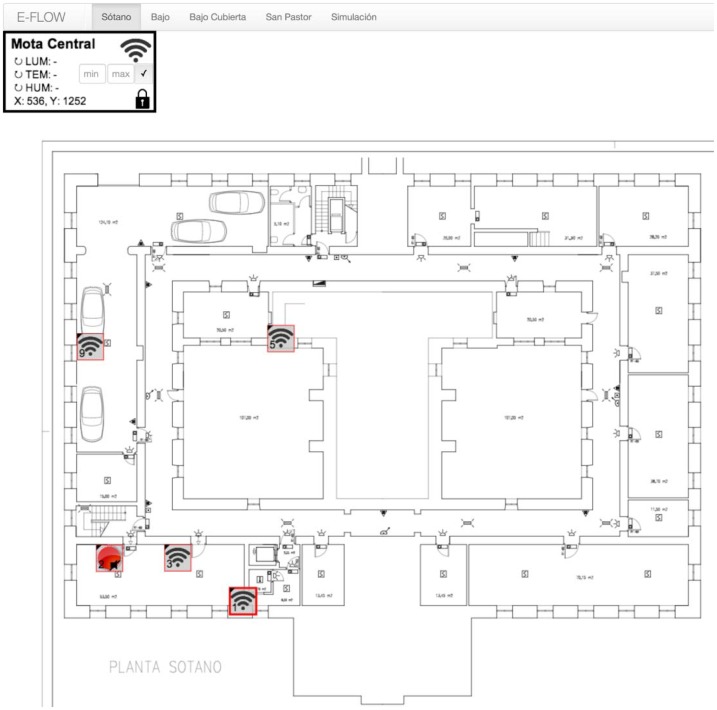
Implementation of the front-end.

**Figure 5.**
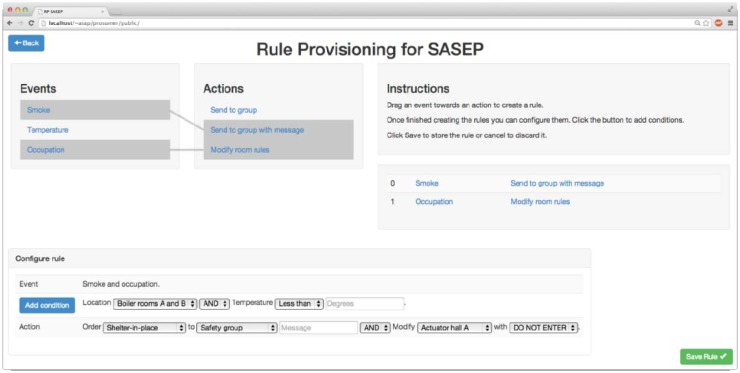
Rule-provision module.

The interface for collecting information from sensors and modifying actuators' states is presented in Figure 4. The RPM is also implemented in Nodejs. As Figure 5 shows, the graphical representations of rules composed of events, conditions and actions are dragged by the user, dropped onto his whiteboard, and the users connect according to the restrictions of a procedure template that is being used.

For example a user can configure an event “temperature”, a condition “location: boiler room AND temperature MORE than 70 degrees”, and action “Order shelter-in-place SEND an alarm to safety group AND modify actuator of building A”. The complexity of atomic interfaces of actuators/sensors are hidden to the rule creator, but implemented by the RPM; so the RPM applies rules by the means of HTTP-REST based events including the identifiers explained in Section 4.

Regarding the user notification and interaction, we developed a native Android application which implements three messaging modes: a centralized XMPP that works with the MCN, a P2P and cellular network. In the first mode, the application is supported by the Openfire server. In the P2P mode, we have employed the AllJoyn [47] libraries to maintain the messaging capabilities in the case the MCN fails. In the third case the UMTS/3G messaging is used, so users can send and received SMS using the same graphical interface. We have also set visual and audible alerts in order to alert users whenever an emergency alarm is pushed to the mobile device. There are also predefined messages that let users quickly publish some emergency warnings such as: “fire”, “stair blocked” and “help please”.

### 5.2. Deployment

**Figure 6.**
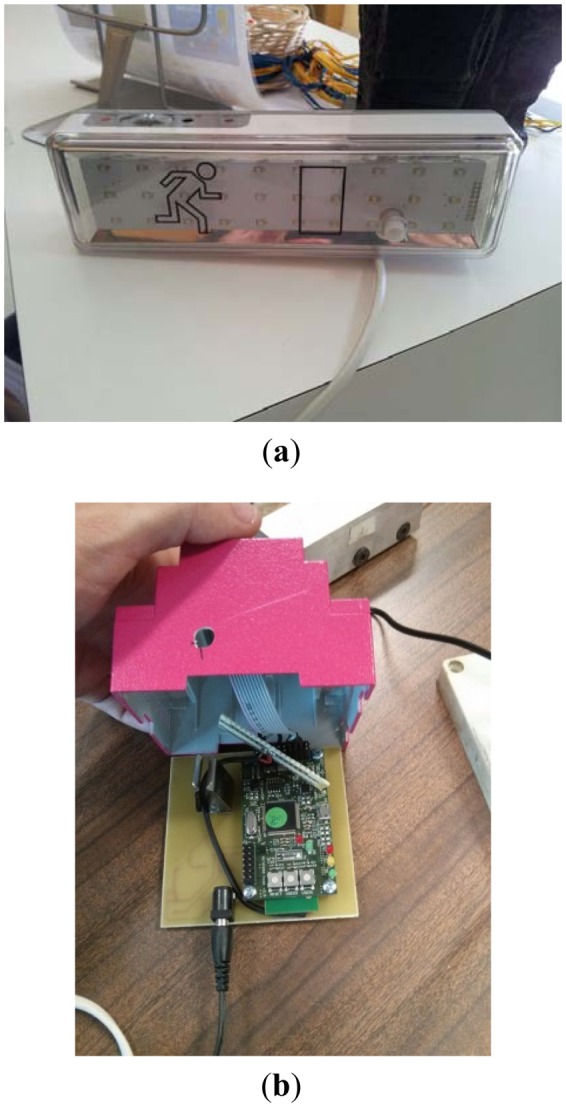
(**a**) Digital evacuation sign (Actuator) (**b**) Wireless Sensor Node containing sensors and actuators.

SASEP has been deployed in a hospital located in Madrid, Spain. In this topology and conditions the average bandwidth between nodes is about 235 Kbps and 10 ms of delay between WSN nodes. Figure 6a shows a WSN node located inside a digital evacuation sign, Figure 6b shows another kind of WSN node that integrates temperature, humidity and occupancy sensors.

**Figure 7.**
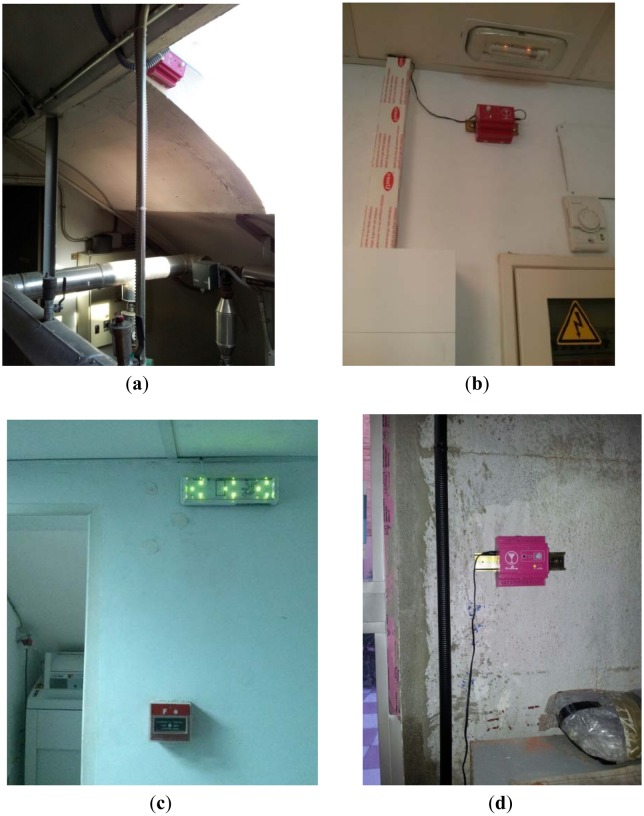
Deployed WSN nodes (**a**) Boiler room (**b**) Servers room (**c**) Emergency paths (**d**) Waste room.

WSN nodes were deployed as it is shown in Figure 7. They were located according to the monitoring needs of the safety staff of the hospital: boiler rooms, the server room, emergency paths and Waste rooms. The specific location of nodes was shown in the front-end application.

## 6. Experimental Validation

This research paper attempts to answer the following research questions:

(1) Which are the benefits of using SASEP to plan an evacuation route?
(2) What is the satisfaction of the people who use SASEP in an emergency situation?

An experimental validation was carried out in order to address these research questions. In this experimental validation, participants were asked to face several emergency situations inside a building. The *test group* managed these situations using SASEP. The *control group* had to face the same emergencies using static evacuation equipment of the building, *i.e.*, labeled exit signs and emergency lights. The shelter-in-place situation was not an option in this experimental validation, so we focused on the situation of imminent evacuation of building for both groups. The researchers that carried out the experiment had little control of the participants who dealt with the emergency situations because they received no external stimulus by researchers. The researchers were limited to take notes on the behavior of participants through observation. This approach is appropriate to replicate the experiment in similar contexts.

### 6.1. Context

The experimental validation were designed, planned, monitored and analyzed by the authors of this research work (hereafter, experts) who have more than five years of experience in computer-based system, end-user interfaces and machine-to-machine technologies.

**Figure 8.**
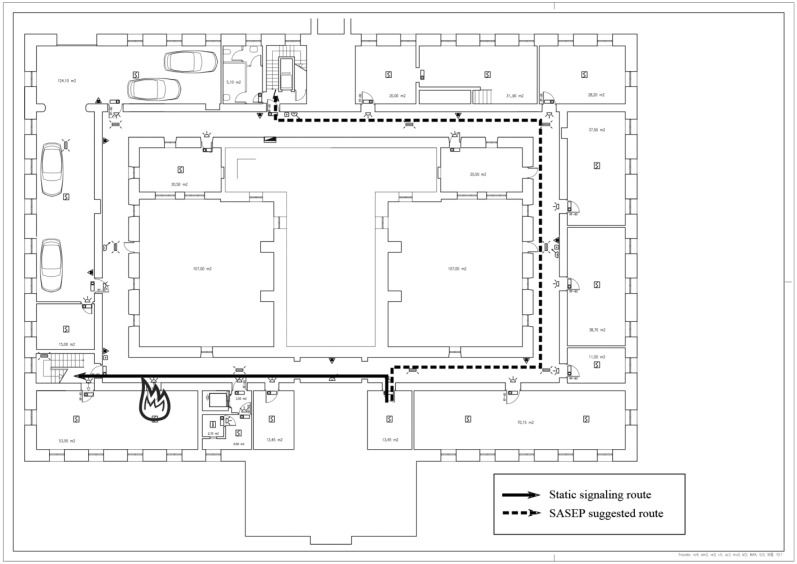
Schema of Situation A.

**Figure 9.**
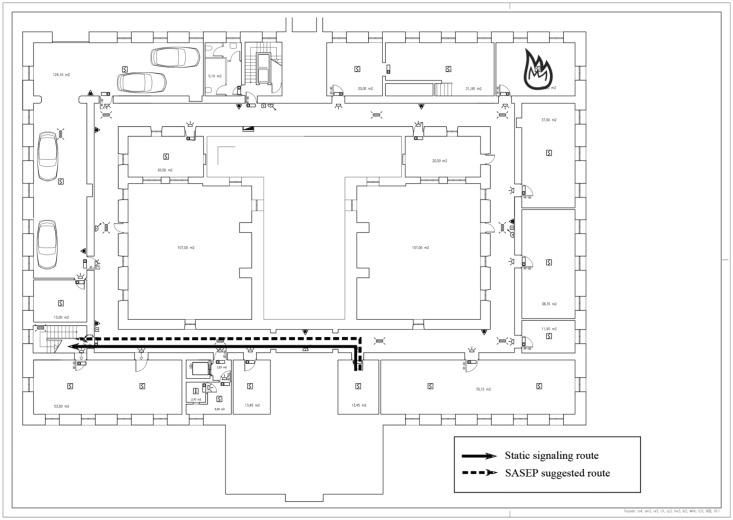
Schema of Situation B.

Thirty two (32) people (hereafter, participants) were involved in the experimental validation, carried out at the hospital. We selected participants with varying ages, good health conditions and prior experience with emergency evacuations. These participants had to deal with two types of emergency situations inside main building of the hospital (See Figures 8 and 9).

In both cases, an emergency is declared in the building and the participant has to reach a safe point out of the building, the distance between the starting point and the nearest exit of the building are very similar, but the difference between the two types of emergency situations were:

Situation A (Figure 8): A simulated threat (such as a fire) is located in the route suggested by the static evacuation plan from the participant position to the nearest exit of the building. In this case the static signs lead to a threat but SASEP suggests an alternative route where there are no threats.
Situation B (Figure 9): The emergency situation consisted of simulate a threat out of the route suggested by the static evacuation plan from the participant position to the nearest exit of the building. In this case SASEP suggested the same route to reach the safe point than evacuation plans using static signs.

An expert followed each participant in the emergency situations described above, and he had no contact with the participant during the experiment. The expert could not answer questions from the participants and the participants knew they could not maintain any communication with the expert.

A full factorial design was used, where the 32 participants were divided in two groups: test and control (see Table 1). The control group, composed of 16 participants, performed two simulations of the emergency situations presented above, using the static evacuation plans to plan the route to reach a safe point out of the building. The test group, composed of other 16 different people, performed the same two emergency simulations, but in this case using *SASEP* to plan the evacuation route. Participant allocation to each group was random.

### 6.2. Plan

**Figure 10.**
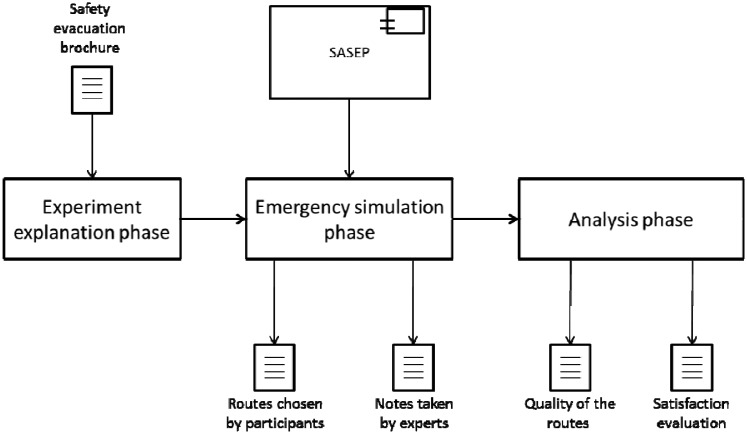
Experiment planning.

The experiment was divided into three phases (as it is seen in Figure 10):

**Experiment explanation phase:** The first phase consisted of the explanation about the simulation of an emergency situation to the participants. They were informed of the basis of the simulation and were given a brochure about recommendations of evacuation. Participants were informed that they will be placed at a point of the building, they will hear an alarm, so then they will have to evacuate following the signs, static or dynamic depending on the situations described in Section 6.1.

**Emergency simulation phase:** The experiment was run by one participant at a time and an expert followed him at a medium distance. The participant was located at the starting point of the experiment and ended when it reached a safe point. Participants used both evacuation plans to find the way to evacuate, depending on the situation they used the static or dynamic to find the route to reach a safe point. The experts took notes about the actions taken by the participants and their reactions to potential threats; they also measured the time they needed to reach a safe point.

**Analysis phase:** The participants at the experiment completed an evaluation survey about the static evacuation plans and SASEP. They were asked about the usefulness, frustration and satisfaction of both systems. In this phase, experts analyzed data collected throughout the experiment.

### 6.3. Data Collection

The information collected to address the first research question (*Which are the benefits of using SASEP for planning an evacuation route?*) was the following:

- Information about the routes chosen by the participants as a way to evacuate. Three experts managing the experiment evaluated separately all the routes followed by the participants at the emergency simulation phase and assessed the quality of those routes. The quality of a route is the level of closeness to the optimal route. The median of a route quality was used for its analysis and a Cohen's Kappa test (squared weights) was performed in order to check the agreement among the evaluations of the three experts; the result was positive indicating that there was a substantial agreement (k = 0.73).
- Time required to reach a safe point. Experts measured in seconds the time took by participants to reach a safe point since the emergency situation started.

The information collected to address the second research question (*What is the satisfaction of the people who use SASEP in an emergency situation?*) consisted of the subjective evaluations of the participants about SASEP and static evacuation plans. The participants were asked about their satisfaction, relevance and frustration levels using both systems. This information was obtained through an anonymous survey completed at the end of the *emergency situation phase* and analyzed by the expert in the *analysis phase*. The issues asked with the survey were made using a Likert scale from 0 to 5, where 0 means low and 5 means high.

## 7. Results

This section presents and discusses the results obtained in the experimental validation. Results are shown following the research questions defined in this paper. The first subsection is focused on the benefits of SASEP, studying in which cases would be an advantage over static evacuation plans. The second and third subsections show the results of satisfaction, relevance and frustration of participants using SASEP and the static evacuation plans.

### 7.1. Benefits of the SASEP for Planning an Evacuation Route

Table 2 summarizes the average quality of the routes chosen by the participants during “Emergency simulation phase”, ranked from 0 to 5. Results show that there are no differences between participants who were evacuated using SASEP and participants who did not use it. A Mann-Whitney U test was performed in order to check whether the use of SASEP increased the quality of the routes; the result of this test was negative meaning that there are no difference with the use of SASEP in both situations. However, it seems that there is a significant difference in Situation A. Another Mann-Whitney U test was conducted to verify that assertion; in this case the results were positive; so it can be stated that there is a statistically significant improvement (p < 0.05) in the quality of the routes chosen by the people who use SASEP to evacuate the building in situation A*.*

**Figure 11.**
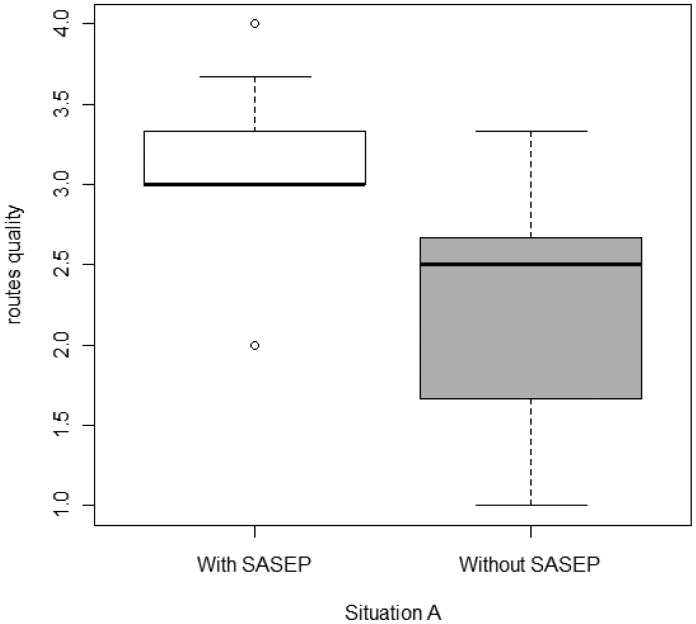
Routes quality for Situation A.

Figure 11 shows the route quality only for “Situation A”. It can be seen that there is a difference between the distributions and the medians.

It should be noted that, in Situation B, the route quality is worse when SASEP is used. Experts took notes during experimentation on the behavior of the participants, their complaints and opinions at “Analysis phase”. They stressed that it was confusing to have two systems, static and dynamic (SASEP), with different evacuation equipment. This is a very important issue to consider in case of deploying a dynamic signs system such as the one presented in this research paper. Information Overload causes confusion in decisions made by humans [48], this is a very important point when talking about emergency situations, so the system should not confuse human and the systems and must be especially clear. It can be concluded that SASEP is a support system that helps positively when the static signs system leads to a threat.

### 7.2. Satisfaction Analysis of the People Who Are in an Emergency Situation with the Use of SASEP

This section presents a brief analysis of the satisfaction perceived by the 32 participants of the experiment using both signaling systems: the classical static signs with predefined evacuation routes and SASEP. The participants were asked about three satisfaction aspects: satisfaction, relevance and frustration. The definitions of these aspects are as follows:

- **Satisfaction** is the overall perception of the usefulness of any system to plan an evacuation route in an emergency situation.
- **Relevance** represents a subjective evaluation about how important or significant are the systems evaluated.
- **Frustration** measures the degree of dissatisfaction when the participants use any of the systems to plan an evacuation route.

The overall evaluation of the participant's satisfaction was positive with an average of 3.06 for the two systems and two situations, which is not very relevant as there are many differences depending on the system used and situations. A deeper analysis must be done in order to get more findings. Table 3 shows the overall scores of the elements evaluated by system and situation.

**Figure 12.**
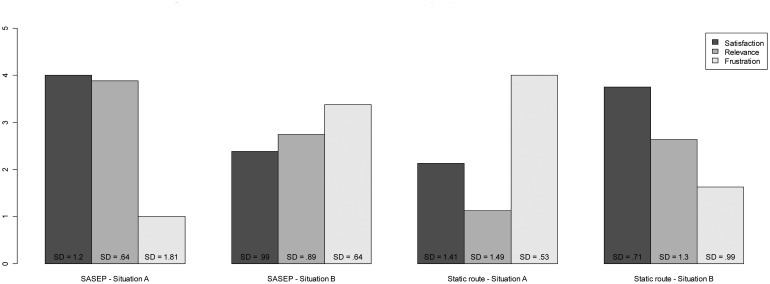
Satisfaction evaluations by system and situation.

There is a great difference in satisfaction assessments depending on systems and situations as it is seen in Figure 12.

Therefore, an analysis of each case will be performed:

- Using SASEP in situation A: In this case there was a threat in the route proposed by the static routes but SASEP detected it and proposed a new route. The participants evaluated positively SASEP with a 4 in average, meaning that they realized that SASEP proposed an alternative route taking them to a safe point. They evaluated the system as very relevant and they seemed little frustrated.
- Using SASEP in situation B: In this case SASEP proposed the same evacuation route that the static evacuation routes implemented by static signs. In this case the participants were dissatisfied with the system because it offered the same evacuation route that static system and they rated it as unsatisfactory and irrelevant. Furthermore as shown in the previous section, participants were overloaded with information of the two systems that led to the same route.
- Using Static predefined routes in Situation A: In this situation the eight participants in the experiment were led by the static signs directly to the threat. Had it been a real case probably many of them could have been injured, but in the experiment were informed that they were approaching a threat, so they had to change the evacuation route, improvising without the help of the signals and using only his intuition. As a result, participants rated the static predefined routes as very unsatisfactory, irrelevant and very frustrating.
- Using Static predefined routes in Situation B: In this case participants used static signs, and there was no threat in the evacuation route, this would be the normal case of use of this system. Participants rated it as satisfactory, relevant and little frustrating.

A high Spearman's correlation (rho = 0.81, p < 0.001) was found between the quality of the routes chosen by participants using SASEP to plan their evacuation plans and their subjective perception of SASEP relevance. It means that participants following the best routes also assessed SASEP relevant to plan their routes. Therefore, SASEP helps to plan good routes to evacuate especially if there is any threat in the static predefined route.

It can be concluded that SASEP helps when there are threats on routes predefined by static signals; however, when there are no threats on the route both systems behave in the same way but the overload of information confuses people creating a lot of frustration. In this case, we propose that in case of deploying a dynamic signal system as SASEP, it should be integrated with static signs (normally already deployed) in order to do not overload and confuse people with massive information; so they can plan the best route to evacuate.

## 8. Conclusions

Evacuation systems are rapidly taking advantage of computer-based systems' capabilities, especially in route optimization, alert notification and automatic decision making. Recent disasters have shown that having clearly defined preventive procedures and decisions is a critical component that minimizes the evacuation hazards and ensure a rapid and successful evolution of evacuation plans. Nevertheless, current state-of-the-art technologies do not consider an end-user solution to simplify how these procedures and decisions are met by specific events, conditions and actions, aligned with the evacuation plan and the technical capabilities of the evacuation system. Our Situation-Aware System for enhancing Evacuation Plans (SASEP) system fills this gap by allowing safety staff to create end-user business rules that technically support these events, conditions and actions while achieving better user situation awareness and flexible data transmission.

(*a*) *Benefits of the SASEP for planning an evacuation route*

A dynamic route guidance system for evacuation does not guarantee that the routes chosen by people are better than static predefined routes system, especially when there is no threat in the static route. However, things are different when there is a threat, such as a fire, in the route marked by the static signals. In this case, a dynamic signaling system, as *SASEP*, offers a better evacuation route option in comparison with static signaling systems.

(*b*) *Satisfaction analysis with the use of SASEP*

As in the previous case, the use of a dynamic route guiding system can lead to misunderstandings and frustrations of people when both systems (static and dynamic) coexist together and there are no threats in the evacuation routes. However, the system is highly valued when there is a threat in the evacuation route, and then the system is assessed as very satisfactory, relevant and little frustrating.

It can be concluded that a dynamic signaling system can be very useful as long as it is integrated with the static signaling system so as to not to create misunderstandings.

(*c*) *Design suggestions for system improvements*

Concerning design suggestion for system improvement we suggest new mechanisms to integrate mobile Pub/Sub brokers, which can enhance the pluggability of new wireless nodes, in specific situations where sophisticated sensing devices are needed (e.g., poisonous gases, or nuclear elements). In our system implementation, the WSN relies on concentrator devices that bridge sensor readings and actions into high level JSON-packets that can be easily processed by web browsers; however, new protocols such as MQTT-S and CoAP could minimize the dependency on these concentrators while minimizing the processing time and keeping data overhead low.

Through our experiments we have found that dynamic signals should be well-located, offer a high illumination level and provide information in a clear way, especially in hospital scenarios, this with the aim of increasing the chances of survival of people with special needs and achieving an optimal route quality and evacuation time. We have also suggest that in case of deploying a dynamic signal system as *SASEP*, it should be integrated with static signs (normally already deployed) in order to do not overload and confuse people with massive information.

## Figures and Tables

**Table 1.** Experiments.

	**Situation A**	**Situation B**
**Control Group**	8 people	8 people
**Test Group**	8 people	8 people

**Table 2.** Quality of the participants' routes.

	**With *SASEP***		**Without *SASEP***	
	Mean	SD	Mean	SD
Situation A	3.08	0.58	2.25	0.77
Situation B	1.67	1.26	2.33	1.28
Total	2.38	1.20	2.29	1.02

Quality scale: 0 = worst; 5= best.

**Table 3.** Summary of participant's satisfaction by system and situation.

	**Satisfaction**	**Relevance**	**Frustration**
**SASEP—Situation A**	4.00	3.88	1.00
**SASEP—Situation B**	2.38	2.75	3.38
**Static route—Situation A**	2.13	1.125	4.00
**Static route—Situation B**	3.75	2.63	1.63

Satisfaction scale: 0 = low; 5= high.

## Notes

**Authors Contributions** Augusto Morales designed the overall architecture of SASEP and contributed to the implementation and deployment of this work. Ramon Alcarria focused in the state of the art and in the situation awareness capability, specifying and implementing the rule-provisioning module. Diego Martin was mainly involved in preparing the experimental validation and processing the results. He also proposed the research questions and the papers structure based on these questions. Finally, Tomas Robles has been responsible for proposing the research topic of situation awareness for evacuation plans, reviewing the work and the paper's preparation.
